# *In vitro* and split-faced placebo-controlled *in vivo* study on the skin rejuvenating effects of cream loaded with bioactive extract of *Indigofera argentea* Burm.f

**DOI:** 10.3389/fphar.2024.1352045

**Published:** 2024-04-04

**Authors:** Tahreem Arshad, Haji Muhammad Shoaib Khan, Kashif-ur-Rehman Khan, Abdulaziz S. Al-Roujayee, Mohamed Mohany, Maqsood Ahmad, Sana Maryam, Muhammad Bilal Akram, Hasnain Shaukat, Umair Khursheed, Mourad A. M. Aboul-Soud

**Affiliations:** ^1^ Department of Pharmaceutics, Faculty of Pharmacy, The Islamia University of Bahawalpur, Bahawalpur, Pakistan; ^2^ Department of Pharmaceutical Chemistry, Faculty of Pharmacy, The Islamia University of Bahawalpur, Bahawalpur, Pakistan; ^3^ Department of Dermatology and Venerology, College of Medicine, Al Imam Mohammad Ibn Saud Islamic University, Riyadh, Saudi Arabia; ^4^ Department of Pharmacology and Toxicology, College of Pharmacy, King Saud University, Riyadh, Saudi Arabia; ^5^ Department of Pharmacology, Faculty of Pharmacy, The Islamia University of Bahawalpur, Bahawalpur, Pakistan; ^6^ APHA-American Pharmacist Association, Washington, DC, United States; ^7^ Faculty of Pharmacy, The Islamia University of Bahawalpur, Bahawalpur, Pakistan; ^8^ Department of Clinical Laboratory Sciences, College of Applied Medical Sciences, King Saud University, Riyadh, Saudi Arabia

**Keywords:** *Indigofera argentea* extract, phenolic quantification, antioxidant, tyrosinase inhibition, UV-protection, cream formulation, *in vivo* studies, skin rejuvenation

## Abstract

The bioactive extracts of traditional medicinal plants are rich in polyphenols and help to rejuvenate skin. The study was designed to assess the skin rejuvenating effects of a stable cream enriched with 4% *I. argentea* (IaMe) extract. The quantity of polyphenols by spectrophotometric methods was TPC, 101.55 ± 0.03 mg GAE/g and total flavonoid content; 77.14 ± 0.13 mg QE/g, while HPLC-PDA revealed gallic acid; 4.91, chlorogenic acid 48.12, p-coumaric acid 0.43, and rutin 14.23 μg/g. The significant results of biological activities were observed as DPPH; 81.81% ± 0.05%, tyrosinase; 72% ± 0.23% compared to ascorbic acid (92.43% ± 0.03%), and kojic acid (78.80% ± 0.19%) respectively. Moreover, the promising sun protection effects Sun protection factor of extract (20.53) and formulation (10.59) were observed. The active cream formulation (w/o emulsion) was developed with liquid paraffin, beeswax, IaMe extract, and ABIL EM 90, which was stable for 90 days as shown by various stability parameters. The rheological results demonstrated the active formulation’s non-Newtonian and pseudo-plastic characteristics and nearly spherical globules by SEM. The IaMe loaded cream was further investigated on human trial subjects for skin rejuvenating effects and visualized in 3D skin images. Herein, the results were significant compared to placebo. IaMe formulation causes a substantial drop in skin melanin from −1.70% (2 weeks) to −10.8% (12 weeks). Furthermore, it showed a significant increase in skin moisture and elasticity index from 7.7% to 39.15% and 2%–30%, respectively. According to the findings, *Indigofera argentea* extract has promising bioactivities and skin rejuvenating properties, rationalizing the traditional use and encouraging its exploitation for effective and economical cosmeceuticals.

## 1 Introduction

Skin health is deliberated one of the principal factors demonstrating overall “wellbeing” and the insight of “health” in humans. Oxidative stress, aging, quantity of melanin, and sun-rays are among the factors affecting the aesthetic aspect of skin. Herein the application of topical formulations and cosmeceuticals are generally considered for the skin rejuvenation (Zouboulis et al., 2019). Several domains employ plant-extracted bioactive materials as nutrition supplements, modern and traditional medicine, synthetic medicine, flavoring, coloring, scents, and cosmetics (Tiwari et al., 2011; Mohammadhosseini et al., 2017). Antioxidant activity is being studied in a wide series of medicinal plant extracts. Antioxidants can be utilized chemically or as plant extracts to reduce oxidative strain, that is involved in destruction (Eghdami et al., 2010; Saeed et al., 2012). The bioactive extracts of medicinal plants contain bioactive phytochemicals, especially polyphenols. Phenolic compounds have redox characteristics due to their antioxidant capacity, that enable them to act as reducing agents, H^+^ givers, metal chelators, and O^2^ quenchers. Appropriate to their potent antioxidant properties, flavonoids are significant, and both the phenolic acids and flavonoids cumulatively contribute to skin rejuvenation by antioxidant, tyrosinase inhibitory, and anti-aging properties (Eghdami et al., 2010; Ahmed et al., 2022a).

Herbal components, when incorporated in the emulsions (w/o and o/w) provide medicinal and aesthetic benefits so getting popularity as cosmeceuticals (Rasul and Akhtar, 2012). Emulsions are one of the best drug delivery system and medication of choice especially for topically used medicaments boosting their bioavailability (Madaan et al., 2014). The use of natural elements in product formulations, which are less dangerous than synthetic ones, is helping cosmetology gain appeal by replacing synthetic components (Isaac et al., 2012). Available in many viscosities, ABIL EM 90 is a translucent liquid used as an emulsifier. A non-ionized surfactant called ABIL EM 90 is used to create w/o emulsions. As per the national formulary, ABIL EM 90 is a methylated linear polymer of siloxane. chlorotrimethylsilane and dichlorodimethylsilane condense to form ABIL EM 90, also referred to as polymethyl siloxane (Raymond et al., 2006). Liquid paraffin, which is a hydrocarbon, changes the consistency and stability of water-in-oil emulsions. Beeswax prevent color change (Mohammad et al., 2015).

An evergreen perennial plant with year-round blossoms called *Indigofera argentea* is a representative of the Fabaceae family and is also stated locally as Hathi, Jantar, and Neel. Another name for it is “wild indigo.” It is found in arid and sandy areas in large quantities. Its oil helps treat wounds, injuries, ulcers, and patchy skin. Its leaves and roots taste bitter (Wariss et al., 2014). The existence of chlorogenic acid, phenols, gallic acid, quercetin, flavonoids, caffeic acid, and ferulic acid favors biological activities and antioxidant potential (Gerometta et al., 2020). It has historically been used to cure skin conditions, headaches, stomach issues, jaundice, and malaria. It is used to alleviate symptoms such as vertigo, fatigue, inflammation, fever, and burns (Javed et al., 2020).

The initial goals of this research were to fabricate a stable cream with bioactive extract from *I*. *argentea* and assess it *in vitro* using a variety of parameters. Stability was measured for 90 days using a variety of metrics, including phase separation, pH, color, and rheology. *Indigofera argentea* was investigated qualitatively using phytochemical analysis. A rheometer was used to measure the viscosity at different temperature time intervals. At different temperatures, occlusive experiments of both active and placebo formulations were carried out to test the cream’s capacity to retain water. The SPF of the extract and the formulation were evaluated at different concentrations. ANOVA and the paired sample t-test were utilized to statistically analyze the collected data and it was hypothesized that findings of our study may be helpful in designing economical and effective cream formulation for skin rejuvenation.

## 2 Materials and methods

### 2.1 Materials

*I.* *argentea*, a member of the Fabaceae family, was used as a material. The entire population of *I. argentea* plants was gathered from the Punjab region of Bahawalpur, Pakistan. After receiving confirmation from the Islamia University of Bahawalpur’s Botany Department that the acquired plant was accurate, it was recorded (under reference no. 43). Beeswax (Merck China), ABIL EM 90 (Franken chemicals, Gebinde), Paraffin oil (Merck KGOA Darmstadt, Germany), and 2,2-diphenyl-1-picrylhydrazyl (DPPH) (Sigma Chemical Co., United States) were employed in this research.

### 2.2 Preparation of the Crude extract of *Indigofera argentea* (IaMe)

After being thoroughly cleansed with tap water, the entire *I*. *argentea* plant was dried in the shade and roughly pulverized. For 72 h, each plant coarse powder was immersed in a 70% aqueous methanolic mixture. The solution (70%) was prepared by taking the 70:30 ratios of methanol and water, respectively. Methanol was selected for its efficient extraction of bioactive compounds from the plant material. This solvent system is frequently reported for the extraction of wide range of phytochemicals, especially the phenolics and flavonoids, which were interested compounds due to their established antioxidant and anti-aging properties. Additionally, water was to ensure the safety of the extraction process by reducing the volatile nature of methanol. The soaking ingredients were first strained via a muslin and Whatman filter paper No. 1 to get filtrate. Once again, the residual material was immersed in a 70% aqueous methanolic mixture. After 3 days of processing, the material was strained, and the residual mass was immersed in 70% aqueous methanol for a third time. There were three executions of the process. A Rotary Evaporator (Heidolph Laborota 4,000 efficient, Germany) was then used to concentrate the filtrate at 40°C. In the end, dense and thick pastes of extract were obtained and the concentrated extract was stored at 4°C (Jabeen et al., 2009).

### 2.3 Phenolic quantification of IaMe extract

Polyphenols are the major bioactive contents in medicinal extracts and cosmeceutical formulations due to their antioxidant, anti-ageing, and anti-tyrosinase effects (Ahmed et al., 2022a). So, the IaMe extract was characterized by the quantity of phenolic compounds by spectrophotometric and HPLC-PDA methods.

#### 2.3.1 Total phenolic content

Using the Folin and Ciocalteu reagent, the extract’s total phenolic content was calculated. Using a UV-visible spectrophotometer at 765 nm, readings of the standard (varying concentrations of 10, 20, 40, 80, 100, and 200 μg/mL in methanol) and samples (1 mg/mL in ethanol) were collected against a blank reagent. The Folin and Ciocalteu reagent was combined with water and the test sample. Sodium carbonate saturated solution (1 mL) was poured into the mixture, while the volume was then raised to 3 mL with distilled water and stayed in the dark for 30 min and centrifuged. Gallic acid standard curves were used to calculate the amount of phenol per gram of dry plant material (Sajid-ur-Rehman et al., 2023).

#### 2.3.2 Total flavonoid contents

The total flavonoid content (TFC) of test sample (I mg/mL in ethanol) was evaluated by Park’s approach with certain modifications. In this technique, 0.3 mL of IaMe extract and 0.5 mol/L of sodium nitrate were combined with 0.1 mL of 0.3 mol/L aluminum chloride hexahydrate. 3.4 mL of 30% methanol was then added, and absorbance was then measured at 506 nm. The standard curve of quercetin (10, 20, 40, 80, 100, and 200 μg/mL in methanol) was obtained and TFC was displayed as mg quercetin equivalent/g of the sample’s dry weight (Ahmed et al., 2022b).

#### 2.3.3 Phenolic quantification by HPL-PDA method

The plant extracts were weighed on analytical balance and solubilized in mobile phase. A Waters liquid chromatograph outfitted with a model six hundred solvent pump and a 2,996 photodiode array detector (PDA) was utilised to perform HPLC analysis. Empower v.2 software, available from Waters Spa in Milford, Massachusetts, United States, was utilised to acquire data. The separation was performed using a C18 reversed-phase packing column (Prodigy ODS (3), 4.6 mm × 150 mm, 5 μm; Phenomenex, Torrance, CA, United States), which was thermostated at 30°C ± 1°C using a Jetstream 2 Plus column oven. The mobile phase was A (acetonitrile +3% acetic acid) and B (Milli-Q water +3% acetic acid) that were used in a ratio (93:7, v: v). The concentration of the samples was set at 1 mg/500 µL. Every sample underwent a 30-s vortex, a 15-min sonication, and a 10-min 10,000 g centrifugation. For analysis, 20 µL of the supernatant were put into the HPLC apparatus. The wavelength range for the UV/Vis acquisition was 200–500 nm. For every compound, the quantitative studies were completed at the maximum wavelength. A 20 μL injection volume was used. Using Biotech DEGASi, mod. Compact (LabService, Anzola dell’Emilia, Italy), the mobile phase was immediately degassed online (Saleem et al., 2020).

### 2.4 Biological activities of IaMe

After the phenolic characterization of the extract, the biological characterization was conducted using various *in vitro* assays, including antioxidant (DPPH) assay, tyrosinase inhibitory assay, and determination of sun protection factor of extract. These investigations were performed to determine the bioactive nature of the IaMe extract and its selection for use in cream formulation.

#### 2.4.1 Determination of antioxidant activity

DPPH was utilized to assess the extract of IaMe’s capacity to scavenge free radicals. In short, 50 mL of methanol were mixed with 0.2 mg/mL of DPPH. Various amounts of this solution (2 mL) were poured in to 1 mL of the extract (0.5 mg/mL) in ethanol. After a good shake, the mixture was stored at room temperature for 30 minutes. A spectrophotometer was employed to get absorbance at 517 nm wavelength. Ascorbic acid was utilized as the reference, and experiment were performed three times to reduce error. The given equation was utilized to find the % DPPH scavenging effect (Dilshad et al., 2022).

$$\text{Inhibiton }\left(\%\right)=\left(\frac{\text{Abs}.\;\text{of}\;\text{control}-\text{Abs}.\text{of}\;\text{test}\;\text{solution}}{\text{Abs}.\;\text{of}\;\text{control}}\right)\times 100$$

#### 2.4.2 Tyrosinase inhibition assay

The technique was applied to sample (0.5 mg/mL) using kojic acid as a (+) control for anti-tyrosinase assay. The end volume of the reaction blend was 100 μL. A total of 40 μL of 100 mM phosphate buffer, 20 μL of mushroom tyrosinase enzyme, and 10 μL of 0.5 mM research material were poured into the 96-well plate. The mixture has been pre-incubated for 5 min at 37°C. After incubation, 30 μL of 10 mM L-dopamine was poured in as a substrate. The components were blended and incubated for a further 10 minutes. At 490 nm, absorption was measured using a Synergy 96-well plate (Tabassum et al., 2023). Enzyme inhibition was determined by formula.

$$\text{Inhibition}\left(\%\right)=\frac{\text{Absorbance}\;\text{of}\;\text{test}\;\text{solution}}{\text{Absorbance}\;\text{of}\;\text{control}\;}\times 100$$

#### 2.4.3 Sun protection factor

For the analysis of the SPF of both the *I. argentea* extract and active formulation, a spectrophotometer was used (Dutra et al., 2004). The weight of the substance was precisely 1.0 g. The material that had been weighed was added to a 100 mL volumetric flask, and the volume was mounted to 100 mL by adding ethanol (10 g/mL). After 15 min of ultrasonication, this dilution (10,000 μg/mL) was filtered through cotton material, with the first 10 mL being discarded. Next, 5 mL of the aliquot was added to a 50 mL volumetric flask, and ethanol (2000 g/mL) was used to raise the volume to 50 mL. Using ethanol as a blank, absorption spectra of the samples were taken at intervals of 5 nm, ranging from 290 to 320 nm. The SPF of the extract and the active formulation at various strengths (10,000 μg/mL, 2000 g/mL) was computed using Mansur’s equation (Mansur et al., 1986).

$$SPF=CF\;x\text{E}\text{E}\left(\lambda \right)\;x\;I\;\left(\lambda \right)x\text{Abs}\left(\lambda \right)$$

Where EE (λ) is the erythmogenic effect of light with wavelength λ, Abs (λ) is the spectrophotometric absorbance values at wavelength λ, and CF is the correction factor (10). EE x I (λ) has constant values.

### 2.5 Preparation of the placebo and active formulations

The placebo was prepared using a mechanical homogenizer (without extract). For the placebo preparation, each item was carefully weighed and wrapped in aluminium foil. Using a prepared water bath, the oily phase containing 3% (w/w) ABIL-EM 90, 14% (w/w) liquid paraffin, and 1% (w/w) bees wax was warmed to 75 ± 5°C concurrently. The distilled water-containing aqueous phase was also heated to 75 ± 5°C in the heated water bath. Swirling at 2000 rpm for 15 min, the aqueous phase (qs. add 100% (w/w)) was added to the oily phase drop by drop. Following 10 minutes, the speed of stirring was lowered to 1,500 rpm for 10 minutes, and then to 1,000 rpm for an additional 10 minutes. 500 rpm was the speed at which the homogenizer was run until complete homogeneity was achieved. After that, the emulsion was let to cool to room temperature. A white-colored homogeneous placebo was prepared for the ensuing studies, split into four parts, and kept at 8°C, 25°C, 40°C, and 40°C+75% RH. In the case of active formulation, the same procedure was repeated. To create the active formulation, which included 4% of the *I. argentea* extract, the aqueous phase and oily phase were combined under the identical circumstances as the placebo (Huma et al., 2020).

### 2.6 *In vitro* assessment of w/o emulsion

The w/o type emulsion was evaluated *in vitro* in the faculty of Pharmacy’s *in vitro* cosmetic lab. Several experiments were conducted on both the active and placebo formulations to characterise the w/o emulsion.

#### 2.6.1 Organoleptic examination

Several aspects such as liquefaction odor, phase separation, and color of both the placebo and active emulsions that were maintained at altered temperatures (8°C, 25°C, 40°C and 40°C+75% RH) were assessed at 0-day, 1 day, 2 days, 3 days, 7 days, 14 days, 21 days, 28 days, 45 days, 60 days, and 90 days.

#### 2.6.2 Morphology

Scanning electron microscopy studied surface morphology (SEM) of IaMe extract loaded w/o cream. The loaded w/o cream have been applied to a double-sided tape previously protected on copper stubs and platinum coated and then examined.

#### 2.6.3 pH analysis

The digital pH meter was used to measure the pH of the placebo and active emulsions at various temperatures (8°C, 25°C, 40°C, and 40°C+75% RH) at 0-day, 1 day, 2 days, 3 days, 7 days, 14 days, 21 days, 28 days, 45 days, 60 days, and 90 days.

#### 2.6.4 Spreadability

The spreadability was estimated by utilizing the technique described by (Chaudhary and Verma, 2014). A round plate is centered with 0.5 g of the formulation, and another glass slide is set on top of it. 500 g of weigh was allowed to remain on the superior glass slide for 5 min then the circle’s diameter was measured.

#### 2.6.5 Rheology

The rheology was done on both placebo and active formulations by employing a rotational rheometer model (DVIII Ultra, Brookfield, United States) at different temperatures throughout a 90-day research period. The factors studied were viscosity, shear rate, and shear stress. For the estimation of attained data, the software Rheocalc V.2.6 was employed (Khan et al., 2023a).

#### 2.6.6 Occlusion studies

The beaker was poured with 50 mL of water and shielded with filter paper. 200 mg of the sample were equally spread on the filter top using a spatula. A beaker with filter paper on top serving as the reference sample had no sample added. The samples were stored at 25°C and 40°C for a whole day. The samples were weighted 24 h later to measure the volume of water spent on evaporation (water flow via filter paper). The following equation was used to get the occlusion factor F (Montenegro and Santagati, 2019).

$$F=\left[\left(A–\;B\right)/A\right]\times 100$$

Where, A and B represent the water loss with and without no the sample (reference), respectively. There is no occlusive influence when the occlusion factor is 0 and the occlusion factor of 100 shows the maximum occlusive impact.

### 2.7 *In vivo* human studies

#### 2.7.1 Study design

The current study design was chosen to be prospective, single-blinded, and placebo-controlled after obtaining ethical permission from The Islamia University Bahawalpur’s Pharmacy Research Ethics Committee under reference number 183. For this investigation, a total of twenty-six healthy female volunteers divided into two groups were used; they ranged in age from 20 to 40 and had Fitzpatrick skin phototype III. All the test participants received thorough material outlining all the crucial study-related events. Each test subject signed a written informed consent form. Women who had normal skin on their forearms and faces were chosen as volunteers. The current study did not include participants who smoked, had skin conditions, showed symptoms of or a history of hypersensitivity, or had any other conditions that might have influenced the results. Throughout the trial, it was forbidden for all subjects to use any other cosmetics or therapies. Patch tests were performed on a 5 × 4 cm area of the forearm to screen for any possible skin sensitivity to the developed emulsion. Participants in group 1 were given the base (control) and active cream jars, which were marked “left” and “right,”, respectively, indicating the corresponding side of the face (test areas) where the formulation was needed to be applied. Similarly, the participants in group 2 were given jars of active formulation (left) and placebo (right). Hence, as a control, the left cheek of the face was chosen. For 12 weeks, the emulsion was used twice a day (morning and evening) after cleaning the face. Throughout the study (12 weeks), measurements of skin parameters were planned after 2 weeks (15 days), with the baseline reading taken at 0 h. Volunteers were requested to wait 30 min on each measurement day before obtaining readings for different skin factors so that the skin could adjust to the indoor environmental conditions of 25°C ± 1°C and 45% ± 2% RH (relative humidity) (Mohammad et al., 2018).

#### 2.7.2 Skin melanin and erythema assessment

The levels of skin melanin and erythema were measured with a non-invasive probe called Mexameter^®^ MPA 5, which is an economical and simple tool. It works on the law of light absorption and reflection. The light emitted by the mexameter probe is reflected by the skin and the receiver (Khan et al., 2023b).

#### 2.7.3 Moisture assessment

The moisture level was determined by using a CM825 corneometer with a MPA5 multiprobe adopter (Courage and +Khazaka electronics). It is designed on the law of capacitance, which determines the dielectric constant of skin. Variations in skin moisture level result in a alteration in the dielectric constant of skin, which is noticed on a corneometer (Khan et al., 2023b).

#### 2.7.4 Skin sebum assessment

The amount of sebum in both cheeks was assessed using Sebumeter MPA 5, a photometric device. By gently pressing a specific opaque plastic tape against the skin for 30 s, Sebumeter, which has an area of 64 mm^2^, captures sebum. The opalescent layer turns transparent upon contact with sebum lipids. The amount of sebum (g sebum/cm2) on the skin surface is shown by the following increase in the opaque tape’s transparency value (Towersey et al., 2021).

#### 2.7.5 Skin elasticity assessment

The cutaneous elasticity of the skin was determined using an Elastometer^®^ E25. Negative pressure was employed as the measuring principle to draw the superficial skin into the measuring hole. Without touching anything, the penetration depth is measured for 6 s. In the display, the elasticity is shown as a percentage (Ali et al., 2019).

#### 2.7.6 Visioface studies with visioface^®^1000D

VisioFace was used to acquire 3D face images (high-resolution photographs) for the evaluation of visible facial skin roughness, skin pores and smoothness. This facial light booth is equipped with a photographic system with an 18-megapixel digital camera that is controlled by a computer. A total of 210 white LED diodes were used to evenly illuminate the exposed face skin region during each visit of the volunteer to take digital high-resolution pictures of the volunteer. With the help of the incorporated research software, it was feasible to computerize the photo processing (Qureshi et al., 2022).

### 2.8 Statistical analysis

In this study, gathered data from various aspects of *in vitro* study (*in vivo* studies, pH, rheology) were evaluated statistically by employing SPSS 20.0. Two-way ANOVA employing LSD test and paired sample t-test were employed to analyze the change of temperature and time for both placebo and active formulations.

## 3 Results

### 3.1 Phenolic quantification of IaMe extract

**FIGURE 1:**
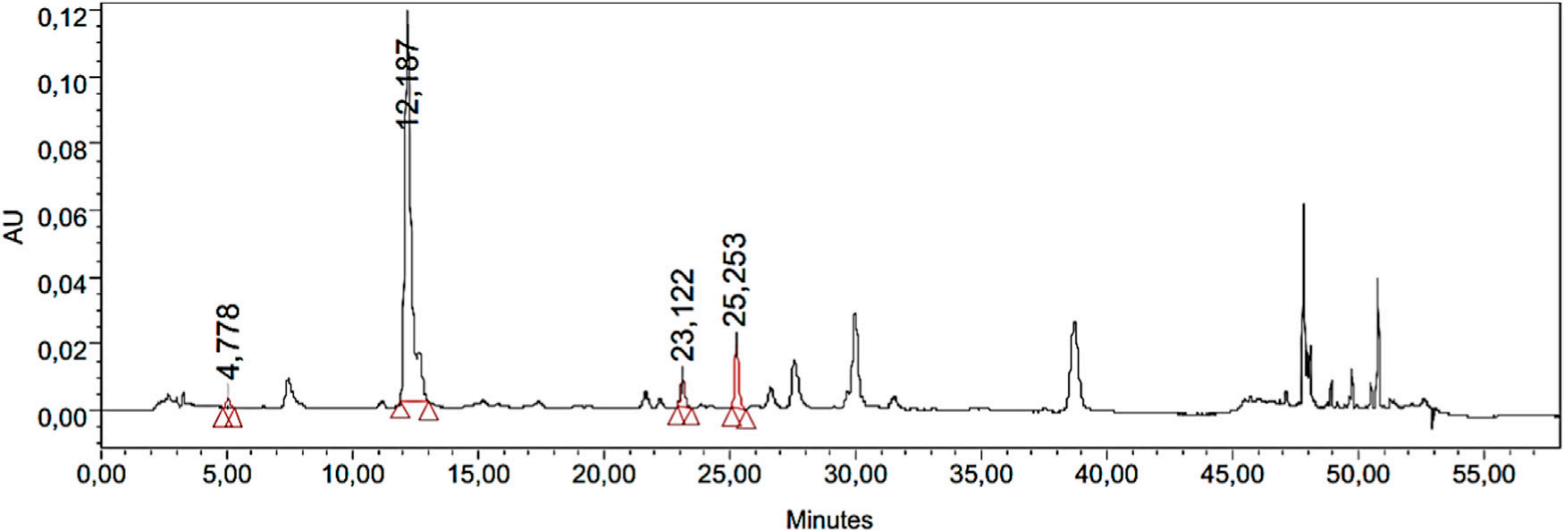
Chromatogram obtained from HPLC-PDA analysis of IaMe showing the presence of four phenolic compounds at different retention times.

The significant quantity of phenolic contents was determined by spectrophotometric methods (TPC; 101.55 ± 0.03 mg GAE/g IaMe and TFC; 77.14 ± 0.13 mg QE/g IaMe). The extract was further investigated for the quantity of individual phenolic compounds with more sophisticated technique (HPLC-PDA). The analysis showed various peaks in HPLC chromatogram, and four peaks were matched with the standards of gallic acid, chlorogenic acid, p-coumaric acid, and rutin at the retention times of 4.7, 12.1, 23.1, and 25.2 min respectively as shown in Figure 1. It revealed the quantity of phenolic compounds as; gallic acid; 4.91, chlorogenic acid; 48.12, p-coumaric acid; 0.43, and rutin; 14.23 μg/g. The detection wavelengths of the mentioned compounds were 271, 324, 309, and 256 nm respectively and the results of the analysis are presented in Table 1.

**TABLE 1:** Quantification of phenolic contents and biological activities to reveal the bioactive nature of the extract of *Indigofera argentea*.

Sample code	Phenolic quantification by spectrophotometric technique			
IaMe	TPC (mg GAE/g died IaMe)		TFC (mg QE/g dried IaMe)	
	101.55 ± 0.03		77.14 ± 0.13	
	Phenolic quantification by HPLC-PDA (µg/g)			
	Gallic acid	Chlorogenic acid	p-Coumaric acid	Rutin
	4.91	48.12	0.43	14.23
	Biological activities			
	DPPH IaMe	DPPH ascorbic acid	Tyrosinase IaMe	Tyrosinase Kojic acid
Inhibition (%)	81.81 ± 0.05	92.43 ± 0.03	72 ± 0.23	78.80 ± 0.19
IC50 (µg/mL)	31.34 ± 0,.45	19.28 ± 0.80	26.03 ± 34	17.40 ± 0.81

### 3.2 *In vitro* biological activites of IaMe extract

After finding the significant quantity of bioactive (phenolic) compounds the extract was investigated for *in vitro* biological activities to reveal its effect on biological system of interest. The determination of antioxidant property by DPPH assay revealed 81.81% ± 0.05% inhibition of DPPH free radicals, which was significant compared to the standard (ascorbic acid; 92.43 ± 0.03). The tyrosinase inhibitory activity of IaMe showed the value of 72% ± 0.23%, which was also significant compared to the standard tyrosinase inhibitor (kojic acid; 78.80 ± 0.19).

### 3.3 Sun protection factor (SPF) of IaMe extract and formulation

SPF of *I. argentea* extract (IaMe) and its cream formulation at the two concentrations (10,000 μg/mL, 2000 μg/mL) were estimated and presented in Table 2. The extract showed the significant value of SPF; 20.53 and 12.40 at the concentrations of 1 g and 0.02 g. The formulation also showed SPF value (10.59) greater than 10.0 at the dose of 1 g, while the value was 3.43 at 0.02 g dose.

**TABLE 2:** SPF of IaMe extract and its cream formulation at different concentrations.

Sr.No.	Wavelength λ (nm)	EE×I Normalized	SPF determination			
			Extract (10000 μg/mL)	Extract (2000 μg/mL)	Active cream (10000 μg/mL)	Active cream (2000 μg/mL)
1	290	0.015	0.19	0.17	0.09	0.09
2	295	0.0817	0.89	0.58	0.49	0.08
3	300	0.2874	5.50	3.57	2.91	0.89
4	305	0.3278	7.62	4.16	3.51	1.27
5	310	0.1864	3.89	2.87	2.46	0.97
6	315	0.0837	1.99	0.91	0.79	0.12
7	320	0.018	0.45	0.21	0.34	0.01
	SPF value		**20.53**	**12.47**	**10.59**	**3.43**

Bold values = the sum of the column, representing the SPF.

### 3.4 Organoleptic assessment of formulations

**FIGURE 2:**
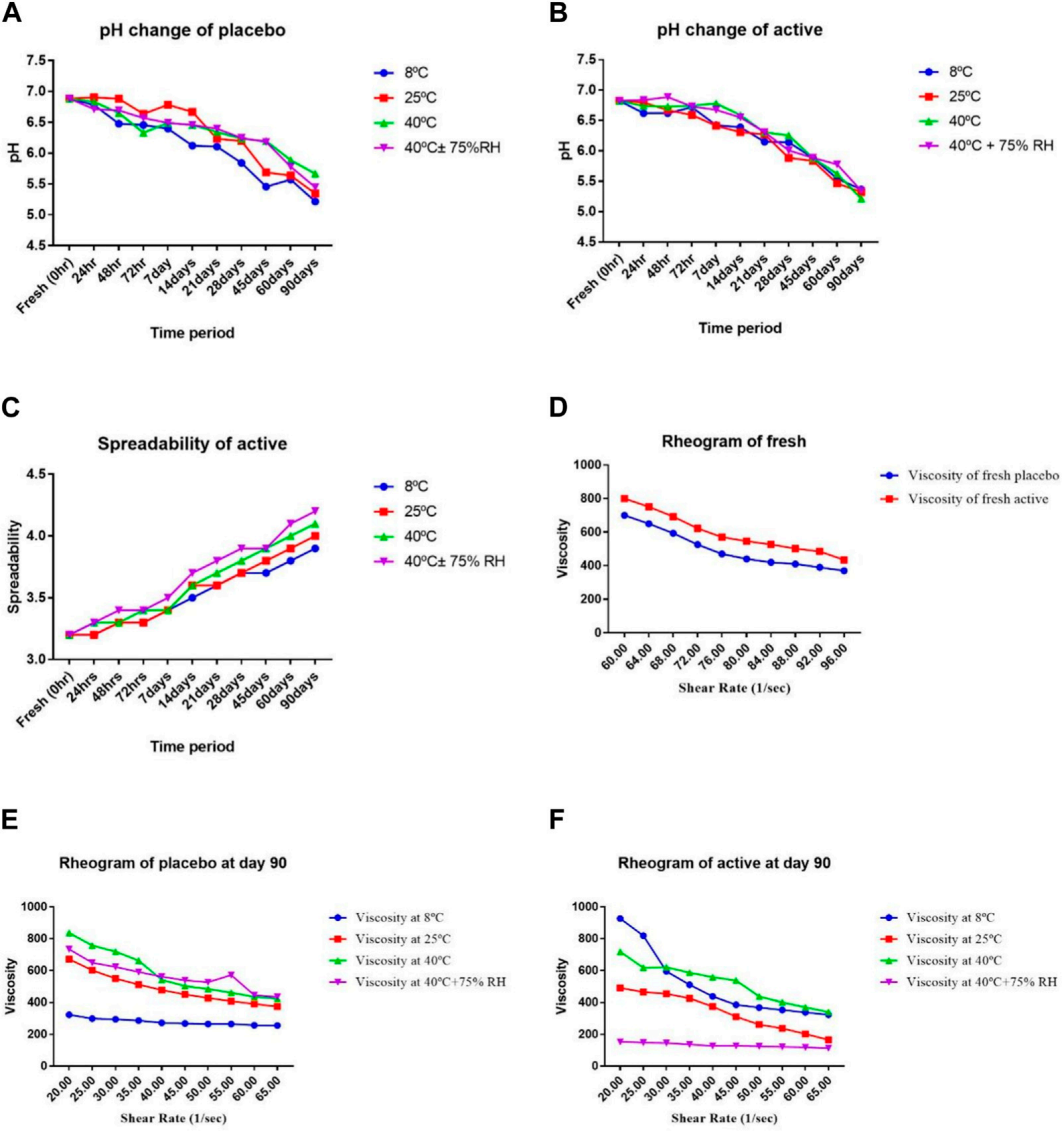
**(A)** pH of a placebo, **(B)** pH of active, **(C)** spreadability of active, **(D)** rheology of fresh, **(E)** rheology of Placebo (90th day), **(F)** rheology of active (90th day), at different temperatures and times.

**FIGURE 3:**
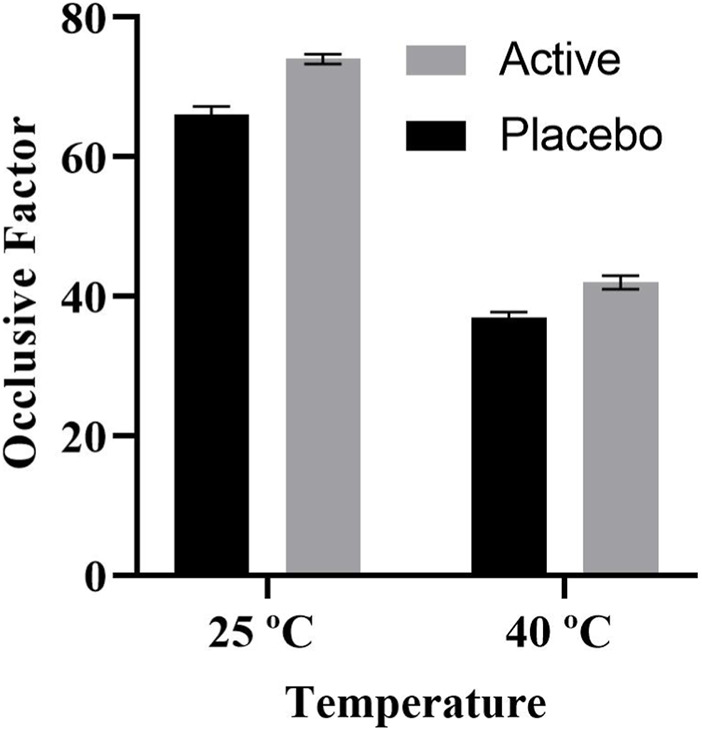
Occlusive factor of both placebo and active at 25°C and 40°C.

**FIGURE 4:**
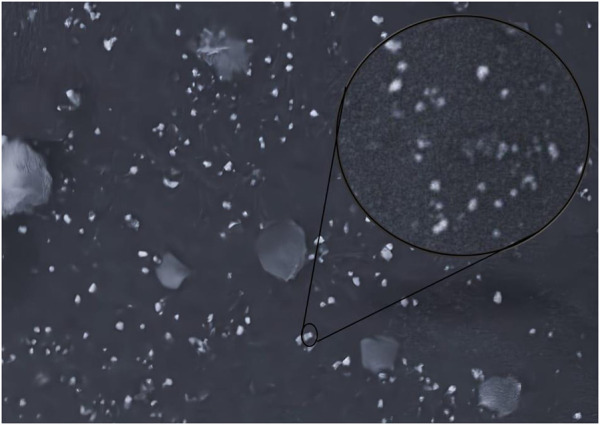
Scanning electron microscopy of IaMe extract loaded cream.

The active and the placebo (without extract) emulsions were retained at various storage settings. Both emulsions were assessed for odour, phase separation, color, and liquefaction for 90 days as indicated in Table 3. Over the course of the study’s 90 days, no formulation’s colour changed while little phase separation and liquefaction were observed on 90 days at 40°C and 75% relative humidity (RH). The gathered data of the pH, spread ability, and rheology were read at altered temperatures as displayed in Figure 2. The pH of the active and placebo decreased slightly over the course of the study’s 90 days while spreadability increased with time and rheology exhibited non-Newtonian and pseudo-plastic characteristics. To assess the water retaining potential of cream, occlusive analyses of the placebo and the active formulation were conducted at 25°C, and 40°C. At 25°C, both formulation exhibited greater occlusive potential then at 40°C, as illustrated in Figure 3. In SEM image analysis of the IaMe w/o formulation, clear spherical or near spherical, unilameller, smooth surface water droplets were observed shown in Figure 4.

**TABLE 3:** Organoleptic assessment of fresh formulations and after 90 days.

After 90 Days										
Monitored parameters	Fresh		8°C		25°C		40°C		40°C ± 75%RH	
	P	F	P	F	P	F	P	F	P	F
Color	wt	l.br	wt	l.br	wt	l.br	wt	l.br	wt	l.br
Phase-Separation	NA	NA	−ve	−ve	−ve	−ve	−ve	−ve	+ve	+ve
Odor	−ve	−ve	−ve	−ve	−ve	−ve	−ve	−ve	−ve	−ve
Liquefaction	−ve	−ve	−ve	−ve	−ve	−ve	−ve	−ve	+ve	+ve

Placebo (P), active formulation (F), not applicable (NA), relative humidity (RH), present (+ve), absent (−ve), light brown colour (l.br), (wt) white.

### 3.5 *In vivo* human studies

#### 3.5.1 Skin erythema contents

**FIGURE 5:**
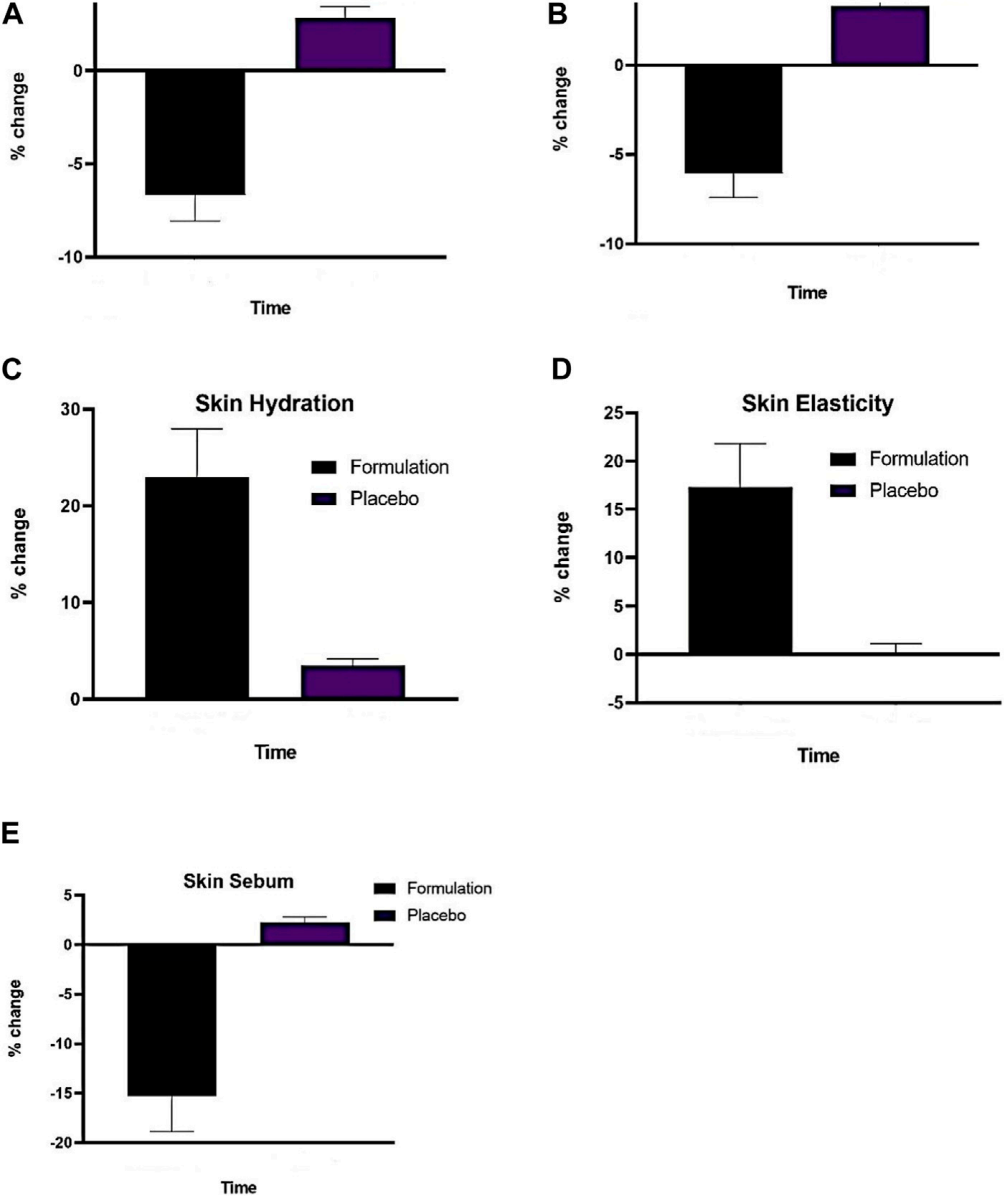
**(A)** Effect of (P) and (F) on skin erythema index applied on human trial subjects **(B)** Effect of (P) and (F), on skin melanin index **(C)**, Effect of (P) and formulation (F) on skin hydration index, **(D)** Effect of (P) and (F) on skin elasticity index, **(E)** Effect of (P) and (F) on skin sebum index (n = 13). Time (12 weeks of study).

As seen in Figure 5A, the placebo in the current experiment showed a random, insignificant increase in erythema. In the case of the active formulation, erythema levels showed a more marked and sustained decline, peaking at −11.77% during the fourth week. It was shown that there was a statistically significant reduction in erythema with these test formulations.

#### 3.5.2 Skin melanin contents

Figure 5B shows how the active emulsion and placebo affect melanin levels based on the current study’s findings. Using two-way ANOVA, it was determined that the melanin level increased somewhat (*p* > .05) after the administration of the placebo. On the other hand, a significant drop in melanin concentration was observed (*p* < .05) over time while using an active formulation from −1.70% (2 weeks) to −10.8% (12 weeks).

#### 3.5.3 Skin moisture content

As shown in Figure 5C, the active formulation in the current experiment showed a substantial increase from 7.7% (week 2) to 39.15% (week 12), whereas the placebo instance showed a significant increase from 3% (week 2) to 6% (week 12).

#### 3.5.4 Skin elasticity contents

The elasticity in the current experiment demonstrated a randomly rising tendency for the test formulation, as seen in Figure 5D. The test formulation’s elasticity value started to increase gradually, steadily, and more noticeably in the fourth week, going from 2% (week 2) to 30% (week 12).

#### 3.5.5 Skin sebum sontents

It was found that, as shown in Figure 5E, the base elevated sebum levels until the 12th week, whereas the AS emulsion test formulations consistently reduced sebum contents at regular intervals for the duration of the study. The results of the ANOVA test demonstrated that beginning in the fourth week and continuing until the end of the experiment, the sebum level increased with base insignificantly (*p* > .05) and reduced significantly (*p* < .05). The sebum values of the base and AS emulsion test formulations varied considerably (*p* < .05) beginning in the fourth week, according to the results of the paired sample t-test.

#### 3.5.6 Visioface studies

**FIGURE 6:**
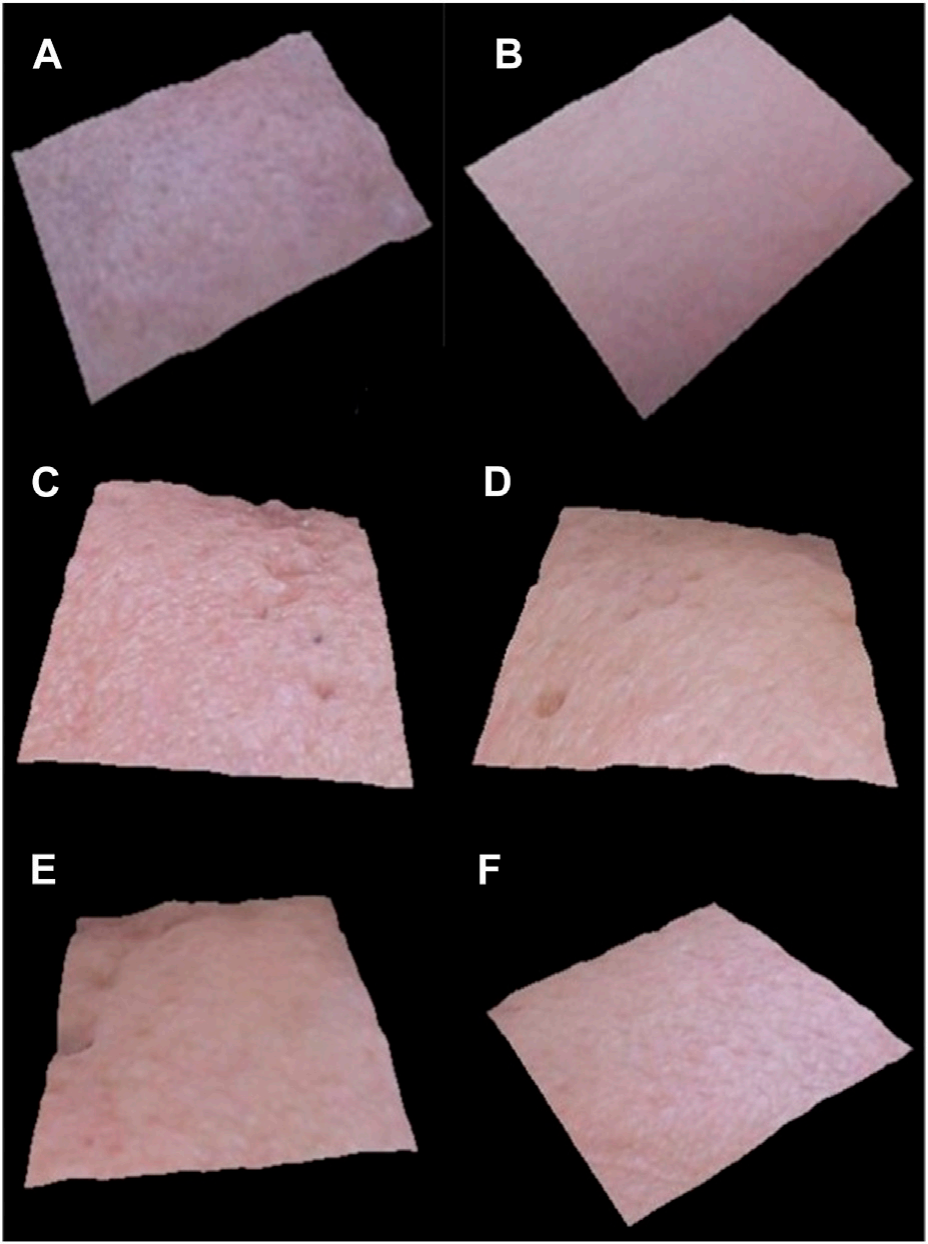
3D facial images of randomly selected volunteers (n = 3), before application of active cream **(A,C, E)** and after 12 weeks of application of active cream **(B,D, F)**.

Figure 6 shows how the VisioFace^®^ uses a high-resolution digital camera to take facial photos and uses white light diodes to take 3D face images that uniformly illuminate the face. It is a tool designed to provide a close-up view of the face and discuss features including fine and large pores, spots, wrinkles, and color variations. Before the base and active formulations being applied, as well as every 4 weeks for the next 12 weeks, 3D pictures were collected. After 12 weeks, the 3D images from the Visioface analysis, as shown in the figures, confirm the beneficial effects of the *I. argentea* extract cream on skin smoothness.

**FIGURE 7:**
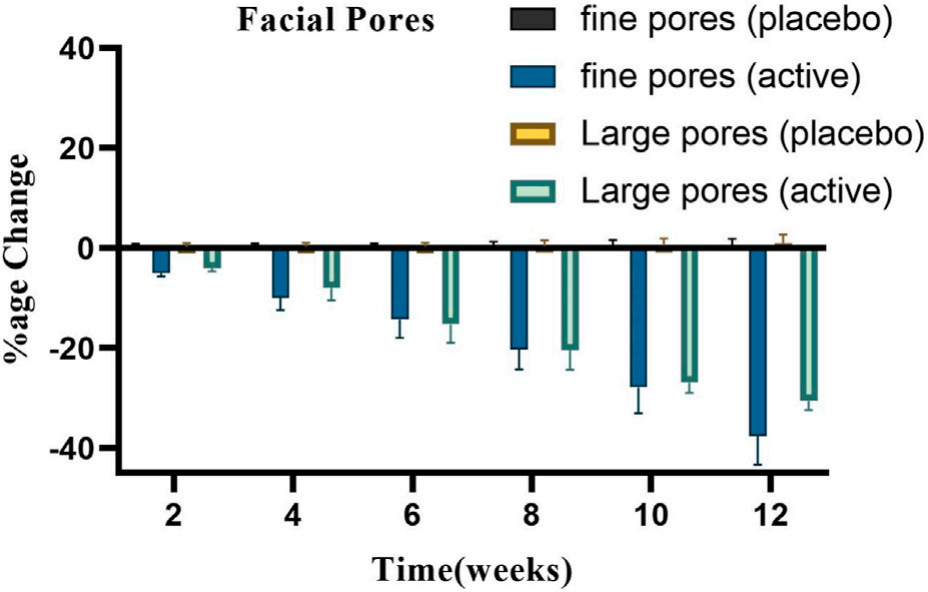
Changes in the percentage of large and fine facial pores.

Figure 7 presents the average % change in the number of pores (both fine and large from three volunteers selected randomly) following the application of the active and placebo formulations at regular intervals for a duration of 12 weeks. After applying the base, the number of fine facial pores showed a slight increase (*p* > .05) up to 0.21% by the conclusion of the study period. After using the active formulation, a substantial (*p* < .05) and consistent decrease in the number of small facial pores up to −37.59% was observed.

Figure 7 also illustrates how basic and active formulations affect the quantity of big facial pores. Large facial pores increased steadily throughout the course of this period, as indicated by the base’s percentage change of 1%; however, the active formulation reduced the number of large facial pores, reaching a reduction of −30.59%. Using ANOVA, it was found that these changes were insignificant (*p* > .05) for the base and significant (*p* < .05) for the active formulation over time. All of the participants who got the active cream treatment saw a decrease in the overall number of large facial pores.

## 4 Discussion

Plants are a valuable source of polyphenols with considerable biological activity including antioxidant and anti-aging activities. Amongst them, phenols and flavonoids have gained more interest (Hagerman et al., 1998). Using a UV spectrophotometer, the standardization processes for quantifying the total phenolic and flavonoid contents were performed from IaMe extract (Table 1). Our conclusions were in agreement with a previous study by Javed et al., 2020, in which they found the quantity of phenolic and flavonoid contents from ethanolic extract of *I. argentea*. Furthermore, it is also revealed in the study that the methanol is slightly more efficacious in the extraction of phenolic contents from the plant (Javed et al., 2020). Due to their redox qualities, polyphenols act as free radical scavengers (Khan K.-u.-R. et al., 2023). The subsequent characterization of extract for phenolic compounds was performed with more sophisticated technique (HPLC-PDA). The detected polyphenols have antioxidant, anti-inflammatory, anti-aging, tyrosinase inhibitory activities documented in the literature. The Quantification of the polyphenolic components directs significant antioxidant potential also. The chlorogenic acid and p-coumaric acid were also described in a earlier study on the ethanolic extract of this plant (Javed et al., 2020), however, their cumulative quantity was greater in the methanolic extract as observed in our study. The *I. argentea* was also containing many phenolic and other bioactive phytochemicals as reported by another study (Arshad et al., 2021). So, the IaMe extract can be considered as a source of bioactive phytochemicals, especially the polyphenols.

Using a spectrophotometer, the DPPH approach was employed to evaluate the extract’s ability to donate electrons or scavenge free radicals (Čanadanović-Brunet et al., 2011). The antioxidant activity of plant extract Indigofera argentea in the methanolic extract at different concentrations was measured with a spectrophotometer and found to be significant compared to the ascorbic acid. The consequences of study were confirmed by findings of a previous study on the ethanolic extract of this plant in which Javed et al. announced the similar radical scavenging potential (Javed et al., 2020). Since phenolic molecules are primarily responsible for the antioxidant potential, the presence of flavonoids and phenolic contents is attributed to these antioxidant activities (WATER, 2013). Table 1 integrates the amount of ascorbic acid as a control to compare the antioxidant capacity of IaMe extract.

Tyrosinase, an essential enzyme of melanogenesis, is a frequent target of cosmetic agents lowering hyperpigmentation of skin. It has a crucial function in altering the production of melanin via tyrosine hydroxylation into dihydroxyphenylalanine (DOPA) and further oxidation into DOPA quinone. Thus, tyrosinase inhibition is the simplest way to attain hypopigmentation (Pillaiyar et al., 2017). The findings are indicative of effective tyrosinase inhibition activity in IaMe extract as shown in Table 1. This activity may also be due to the phenolic and flavonoid contents as well as the quantity of four polyphenolics detected in the study.

Extracts containing phytochemicals including flavonoids, tannins, and phenols offer protection against the sun. The UV spectrophotometer is an easy, rapid, and economical method for determining the SPF *in vitro* of cosmetic products that contain plant extracts (Fatima Grace, , 2014). At 10,000 g/mL and 2000 g/mL concentrations, the extract’s SPF values were 20.53 and 12.47, respectively. At strengths of 10,000 g/mL and 2000 g/mL, the SPF of the IaMe emulsion was 3.43 and 10.59, respectively (Table 2). Since previous studies have shown that plant extract good in phenolic and flavonoid components can penetrate UV rays tremendously and can validate potential natural shades glasses in cosmetic preparations, preventing skin damage, the SPF values can be due to the occurrence of the phytocomponents in the extract (Costa et al., 2015).

The results confirmed that the active formulation had a light brown hue, while the placebo was white. There was no discernible color shift in any of the formulations, as seen in Table 3. This is because, in addition to oxidative deprivation and microbes’ growth, that alter color of emulsion, the antioxidants in the I. argentea extract have natural preservation capabilities. The beeswax employed in the active and placebo formulations can also regulate the colour shift. The emulsifying ingredient used in the production of the active and placebo formulations also stops the colour shift (Mohammad et al., 2015). Table 3 shows that after 90 days at 40 OC± 75% RH, the formulations partially liquefied. Viscosity varies because of liquefaction, which is brought on by variations in temperature and time. As the liquefaction process goes on, the viscosity decreases (Smaoui et al., 2017). Phase separation was not observed over the first 60 days of maintaining the active and placebo formulations at various temperatures. This is because the w/o emulsion was created with good homogeneity. The formulation did not break once the stress was applied because of appropriate homogeneity (Waqas et al., 2010). However, as Table 3 illustrates, there was some phase separation on the 90th day of both the active and placebo formulations, which were maintained at 4°C ± 75% RH.

The skin often has pH fluctuations between 4.0 and 7.0. Skin with a pH of less than 5.0 is considered safer than skin with a pH of more than 5, according on measurements of numerous aspects of the skin, including scaling and moisture (Lambers et al., 2006). (Lambers et al., 2006). As shown in Figure 2 (A, B), the pH of the active and placebo formulations drops from the first day to 90 days. The pH decreases as liquid paraffin breaks down into organic acids and aldehydes (Sharif et al., 2015). A two-way analysis of variance was performed to validate the difference in pH of the active and placebo formulations. The results showed that the pH fluctuation is statistically significant (*p* < 0.05) with respect to temperature and time. A paired sample t-test was employed to evaluate all the active and placebo formulations many times in different storage conditions. The pH levels of the active and the placebo differed significantly. The pH showed a substantial change (*p* < 0.05).

The spreadability index assesses the viscosity estimation, consistency, and ease of application of the active formulation. It is a crucial factor in both client approval and the permanence of emulsion storage. It must remain constant and within the permitted range. If the value of the formulation started to rise endlessly, it might liquefy or break into phases (Shakerardekani et al., 2013). The computed spreadability value for a new formulation was 3.2. As shown graphically in Figure 2C, the rise in readings afterwards 90 days was 3.9, 4.0, 4.1, and 4.2 at altered temperatures. These results suggest that compared to extreme temperatures, a temperature of 8°C increases spreadability less. Spreadability grows with temperature, indicating the stability of the formulations. As we contrast the readings of fresh active with the data from 90 days afterward, there is a rise in the values, but not a significant shift.

All the rheograms showed that viscosity is decreased by increasing the shear rate. Shear stress and viscosity are inversely correlated with shear rate, as Figure D,E,F illustrates. This inverse relationship results from the formulation’s generation of aggregates in the absence of shear stress. These clumps disintegrate under stress, which lowers the viscosity. Systems that shear thin are non-Newtonian. The proportion of the internal phase determines the cream shear thinning behaviour. Oil in water cream has a high flow index because of the low concentration of dispersed phase caused by tiny droplet contact. The viscosity of the external and dispersed segments of the emulsion, the droplet size division, the emulsifier interfacial viscosity and other factors that affect formulation are all described by rheological analysis of a cream. At extreme temperatures, like 40°C and 40°C± 75%RH, the viscosity decreases. Temperature increases improve the flow characteristics of molecules between interfaces, which lowers viscosity. This flow index is connected to a reduction in viscosity in any formulation (Khan et al., 2011). Based on different temperatures and time intervals, a two-way ANOVA showed a significant (*p* < 0.05) change in viscosity for both the active and placebo formulations. The viscosity of the active and placebo formulations was significantly (*p* < 0.05) correlated, according to a paired sample t-test.

The occlusive property of the active cream is a key tool for determining the level of skin moisture. Medication can readily permeate the skin since cream formulations are more occlusive. The occlusive values of the prepared IaMe were estimated; Figure 3 shows the results. Beeswax was a component of the mixture, which contributed to its exceptional occlusive properties (Hagerman et al., 1998). The findings of similar study, emphasized the significance of temperature-dependent occlusive effects in skincare formulations, are consistent with this phenomenon. The study’s findings indicate that some creams function best with their occlusive qualities at lower temperatures, which improves their ability to keep skin hydrated. Conversely, at 25°C and 40°C, the placebo showed less occlusive action, indicating that the benefits of the *I. argentea* cream may be unique to its formulation. This discovery emphasises how important botanical extract is to the cream’s occlusive qualities. Prior research has highlighted the function of components derived from plants, as those in *I. argentea*, in creating efficient occlusive walls (Arshad et al., 2021; Khan K.-u.-R. et al., 2023).

In conclusion, the *I. argentea* cream’s demonstrated temperature-dependent occlusive activity highlights its potential as a flexible skincare product. The distinctive qualities of the botanical extract in contributing to the occlusive component of the cream are highlighted by this reactivity to environmental variables as well as the observed differences from the placebo.

The protective system of the skin is compromised by erythema and inflammation, which can have further detrimental effects such as increased trans-epidermal water loss, elasticity loss, and dryness (Bhat et al., 2022). Finding natural treatments that help reduce erythema and shield skin harm is crucial, though. As seen in Figure 5A, the results showed that, in contrast to placebo, the formulation continuously decreased the erythema index to −11.77% during the duration of the 12-week study. ANOVA statistical analysis with a 5% threshold of significance showed that the change in the erythema index by the formulations was significant (*p*∼0.05) whereas it was negligible (*p* > 0.05) with the placebo. The inflammatory response to is mostly caused by the overproduction of reactive oxidative species and the deterioration of the antioxidant endogenous system associated with chronic UV radiation skin exposure (Leaf-nosed bat and in Encyclopædia Britannica, 2009).

Melanin pigment is the primary cause of skin colour in humans. Plants, fungi, and bacteria are all made of the pigment melanin. Melanin production is carried out by the enzyme tyrosinase (Hariri et al., 2021). Tyrosinase underactivity results in vitiligo, or skin depigmentation, and hair lightening, whereas overactivity leads to an excess of melanin production and hyperpigmentation of the skin. Thus, tyrosinase inhibition may lead to a decrease in the synthesis of melanin. Topical treatments containing both natural and synthetic ingredients are commonly used to treat hyperpigmentation. Green tea, azelaic acid, dioic acid44, licorice, N-acetyl glucosamine, rucinol, and ellagic acid. In this current investigation, the skin melanin content was shown to be higher in the placebo group and declined in the test formulations (Konda et al., 2012). In this current investigation, the skin melanin content was shown to be higher in the placebo group and declined in the test formulations. As shown in Figure 5B, the melanin index dropped sharply after using the formulation, going from −1.78 percent (week 2) to −10.8 percent (week 12). The two-way ANOVA statistical analysis, conducted at a 5% significance level, demonstrated that all formulations significantly altered the skin’s melanin content (*p*∼0.05). However, the influence on the placebo group was not statistically significant (*p* > 0.05). The IaMe extract’s phenolics and flavonoids could be the cause of the drop in skin melanin content. The tyrosinase inhibitory activity of flavonoids may be explained by chelating the enzyme’s active core, which causes the formation of melanin contents (Saewan et al., 2011).

The primary selection criteria for skin care products also consider the degree to which the various layers of skin retain moisture. The tissues that hold moisture in the skin are damaged as we age and the environment changes. If you take 10% off the water content, your skin gets dry and wrinkled and loses its suppleness. Plant extracts with biological properties are replacing synthetic moisturizing agents due to their superior moisture retention capacity and advantageous effects on skin nutrition (Arshad et al., 2020). Skin moisture contents grew progressively after repeated treatment for 12 weeks, according to this study’s measurement of the % change in moisture contents following application of each formulation. There was a discernible increase in the percentage of the emulsion containing plant extract from 7.7 percent (week 2) to 39.15 percent (week 12). At a significance level of 5%, the two-way ANOVA statistical analysis demonstrated that the formulation significantly (*p*∼0.05) increased skin moisture levels, whereas the placebo had negligible (*p* > 0.05) effects. Furthermore, Pro-filagrin is the primary epidermal barrier that keeps the skin’s moisture content constant. It is composed of filaggrin monomer units that are still active and capable of differentiating between free amino acids in the stratum corneum. Maintaining skin hydrated requires these naturally moisturising amino acids, which are hygroscopic when combined with solid ions (Ovaere et al., 2009). Furthermore, the skin moisture index rose compared to the placebo. The IaMe extract includes flavonoids that upregulate filaggrin, resulting in its differentiation and moisturising properties (Rawlings and Lombard, 2012). According to reports, the presence of different plant polyphenols may be linked to this increase in skin hydration because of their potential to reverse the effects of UVR-induced photoaging, which improves skin moisture and blood flow (Javed et al., 2020).

The skin is continuously exposed to UV light, which helps to produce collagenase and raises the risk of inflammatory reactions. Fibroblasts, which are present in the dermis of human skin, contain collagen, which is required to maintain the skin’s elasticity (Varani et al., 2006). A decrease in skin elasticity 50 or a loss of skin elasticity is indicative of skin ageing (Dobrev, 2007). As seen in Figure 5D, the formulation including antioxidant extracts has been found to have a significant impact on skin elasticity, with a percentage shift from 2% (week 2) to 30% (week 12) compared to a placebo’s 3% (week 2).). All test formulations significantly altered the skin’s elasticity (*p*∼0.05), according to the statistical analysis utilising a two-way ANOVA test with a 5% threshold of significance; however, the placebo produced an inconsequential change (*p* > 0.05). The content of phenols and flavonoids in extract is closely associated with the preservation of the skin’s healthy texture (Camejo et al., 2010). Increased elasticity can be attributed to the presence of chlorogenic acid, rutin, and other polyphenols, which produce elastin and collagen to protect the skin from photodamage and free radicals, hence reducing roughness on the skin (Javed et al., 2020).

A natural body fluid produced by humans, sebum keeps the skin hydrated and shields it from pollutants and germs (Zaman and Akhtar, 2013). Excess sebum production and additions result in oily skin, which can trigger acne and seborrhea (Laneri et al., 2021). Overproduction of sebum in human skin has been found to be associated with the pathophysiology of acne (Jović et al., 2017). Herbal treatments are commonly used to treat acne because of the herb’s antibacterial, anti-inflammatory, anti-androgen, and antioxidant qualities. Figure 5E illustrates how the sebum level dropped in this study, with an average percentage shift from −2.89 percent (week 2) to −26.94 percent (week 12). All test formulations significantly altered the amount of skin sebum, whereas the control had no effect (*p* > 0.05), according to a two-way ANOVA statistical analysis with a 5% level of significance (*p*˂0.05). This decrease in skin sebum levels supports the earlier research. The formation of sebaceous glands (excessive sebum excretion) is linked to the -1-reductase enzyme because of the production of dihydrotestosterone. Many phytochemical elements, including sterols and polyphenols, which also help to reduce sebum levels, inhibit the −1 reductase enzyme (Dobrev, 2007).

During a 12-week period, the 3D images acquired via Visioface analysis are essential in giving a visual depiction of the skin’s reaction to the cream administered. These photos provide useful information about the long-term effects of the cream on the skin and can be used to objectively evaluate changes in the texture and smoothness of the skin.After 12 weeks, there was an improvement in the smoothness of the skin, which is consistent with earlier studies showing specific botanical extracts have qualities that affect the texture and health of the skin. Notably, the skin’s surface appears to benefit from the cream containing *I. argentea* extract, giving the appearance of smoother, more refined skin. The possible advantages of using natural extracts in skincare formulas have been highlighted by numerous studies (Čanadanović-Brunet et al., 2011; WATER, 2013).Other studies showed that substances originating from plants have the ability to smooth the skin and linked this impact to the presence of antioxidants and anti-inflammatory agents (Čanadanović-Brunet et al., 2011; Pillaiyar et al., 2017).

Our study’s findings of the antioxidant qualities of *I. argentea* extract may be a factor in the skin texture improvements that have been seen and may be due to the presence of active secondary metabolites, mainly polyphenols possess strong antioxidant potential and hence causes skin smoothness (Javed et al., 2020). In addition, the extended 12-week trial period enables a thorough evaluation of the cream’s effectiveness as shown in Figure 6. The idea that some botanical components may require prolonged treatment to exhibit their full advantages on skin health is consistent with the slow and steady increase in skin smoothness.

## 5 Conclusion

Based on the research findings, it was determined that *I. argentea* could be applied cosmetically and, has promising skin rejuvenation, UV protection properties, and may be economical cosmeceutical. IaMe extract exhibited efficacy by improving skin textures and flaws related to various factors, including skin melanin, elasticity, moisture, and sebum. Additionally, the formulation’s pH and rheology were ideal. The potentially limited sample size and duration of human trials may be limitations of this study. Further research may focus on more comprehensive and diverse clinical trials to validate the efficacy and safety of IaMe formulations across various demographics and skin types. Future research addressing these limitations could provide a more comprehensive understanding of the cream’s efficacy and safety for skin rejuvenation. Multiple emulsions, lotions, microemulsions, and nanoemulsions are other dosage forms that can be generated for additional research to develop economical cosmeceuticals. Tests for *in vitro* and *ex-vivo* permeability of current formulation will be feasible in the future.

## Data Availability

The original contributions presented in the study are included in the article/Supplementary Material, further inquiries can be directed to the corresponding authors.
